# Quantitative systematic review of the transformational leadership style as a driver of nurses' organisational commitment

**DOI:** 10.1002/nop2.1671

**Published:** 2023-03-14

**Authors:** Xiong Haoyan, David Waters, Huang Jinling, Liu Qiongling, Lin Sien

**Affiliations:** ^1^ Nursing Department Affiliated Hospital of Guangdong Medical University, Guangdong Medical University Zhanjiang China; ^2^ Brimingham City University Birmingham UK; ^3^ Orthopaedic Center Affiliated Hospital of Guangdong Medical University, Guangdong Medical University Zhanjiang China; ^4^ Nursing Faculty, Guangdong Medical College Dongguan China

**Keywords:** literature review, nurses, organisational commitment, systematic review, transformational leadership

## Abstract

**Aims:**

To explore the association between nurses' perceptions of their nurse manager's transformational leadership style and nurses' organisational commitment.

**Design:**

Narrative systematic review.

**Data Sources:**

The CINAHL Complete, MEDLINE, PubMed, Business Source Complete, Cochrane Library, along with OpenGrey t were systematically searched for observational studies written in English, between January 2009 and December 2020.

**Review Methods:**

This systematic review is based on the guidelines of the Cochrane Handbook, and PRISMA‐P. Two reviewers independently selected studies. The quality of evidence was assessed using the Joanna Briggs Institute (JBI) Critical Appraisal Tool for Cross‐Sectional Studies.

**Results:**

Seven cross‐sectional studies with 2885 participants were included. Six studies reported that the transformational leadership style was positively related to nurses' organisational commitment, and the remaining study reported a negative association.

**Conclusions:**

Six studies were found that the transformational leadership style is a driver of nurses' organisational commitment. Only one study reported that transformational leadership style negatively associated with acute care nurses' organisational commitment. However, the negative finding is less valid, as the study data indicates that the nurse managers' ineffective transformational leadership style in the acute care unit or the culture influence, which may have influenced the results.

**Impacts:**

It provides the guideline, recommendation, and important evidence to support nursing managers adopting the transformational leadership style to promote nurse retention helping to alleviate the nursing shortage. This is beneficial to the well‐being of the nurse. Meanwhile, this can help the health organisation reducing the cost of nurses' turnover and recruiting new nurses. It is also good for address future ageing population healthcare problem in the long term.

## 1 INTRODUCTION

Nurse shortages are a global problem. In the US, it was predicted that there would be 0.8 million vacant nurse positions by 2020 (Hudspeth, 2013), and 0.59 million were predicted in Europe (Matrix Insight, 2012). Over the next decade, a shortage of 60,000 nurses is expected in Canada, a shortage of 90,000–109,500 is expected in Australia (Buchan et al., 2013), and a shortage of 1.59 million is expected in China (Liu & Yunibhand, 2016). The rapid growth of the older population is also a global problem. The Royal College of Nursing (Great Britain) (2017) has highlighted that ageing populations will worsen the existing nurse shortages. Population ageing can increase the demands on healthcare services, shrink the healthcare workforce (as existing nurses get older) (Beard & Bloom, 2015), and make it more difficult to recruit sufficient young nurses in the future. Moreover, the healthcare demands of the older population will both increase and change, with studies pointing out that many age‐related diseases will increase, particularly circulatory, eye, and blood and blood‐forming organ diseases (Vrhovec & Tajnikar, 2016; Yeo et al., 2016). This highlights that population ageing will increase the healthcare burden and worsen the existing nurse shortage.

To address the nurse shortage issue, a useful strategy is to increase the supply by increasing nurse training places and recruiting more nursing students; the other useful strategy is to ensure that the nurse retention rate is high (World Health organisation (WHO), 2006; Scott, 2017; European Commission, 2015). In recent years, many countries have increased the recruitment and training of new nurses. These strategies have had some positive effects. However, they may not be sufficient to address the predicted lack of nurses in the coming years because of the lack of adequate numbers of faculty to train new students (Duvall & Andrews, 2010) and because nursing is often not very appealing to young people due to it being underpaid (Coombs et al., 2003). Therefore, more focus should be paid to retaining nurses if healthcare organisations hope to address the nurse shortages.

However, the percentages of existing nurses intending to leave their jobs is high (Aiken et al., 2013; National Council of State Boards of Nursing, 2010), and nurses' intention to leave their jobs is directly related to the turnover rate (Takase et al., 2015). Globally, many healthcare organisations' annual nurse turnover rates are as high as 20% (El‐Jardali et al., 2009; Hayhurst et al., 2005). Copanitsanou et al. (2017) highlight that high turnover is an important reason underlying the nurse shortages. Moreover, studies have concluded that.

organisational commitment (OC) is a major factor that can reduce nurse turnover (Meyer & Herscovitch, 2001; Somers, 2009; Takase et al., 2015). Therefore, improving nurses' OC to reduce the turnover rate should be a primary focus.

## 2 BACKGROUND

Meyer et al. (2002) found that effective leadership has a positive influence on OC. Effective leadership skills among nurse managers increase nurses' OC (and leadership skills are also useful more generally in healthcare) (Cummings et al., 2010). Effective leadership can improve the work environment by providing effective organisational support so that nurses' feel more valued in the team and their inner working lives are improved (Al‐Hamdan et al., 2017; Sherman & Pross, 2010). A good work environment can improve job satisfaction and OC, thus helping to retain nurses (Aiken et al., 2013; Copanitsanou et al., 2017; Laschinger & Finegan, 2005; Li et al., 2018). Nurse managers have the responsibility for promoting care, ensuring patient safety, enhancing the quality of nurses' working lives, and managing the change processes related to all nursing care duties (Everson‐Bates, 1992; McNeese‐Smith, 1997; Sullivan & Garland, 2013). Therefore, it is important for nurse managers to adopt an effective leadership style to strengthen OC in order to reduce nurse turnover and to ensure safe and high‐quality care.

The transformational leadership (TFL) style is the preferred leadership style among nurse managers (Ferguson, 2015). Transformational leadership has been the most influential leadership theory in recent decades (Judge & Piccolo, 2004). Transformational leadership (Bass, 1985) is a transformation process in which leaders inspire their followers to have the belief that they can reach exceptional achievements. Transformational leaders*'* long‐term aims are to change and transform employees in their ways of thinking to achieve leadership effectiveness (Bass, 1985; Yukl, 1999). Therefore, the leader's transforming abilities are a key to the work of transformational leadership.

Transformational leadership is comprised of four components (Bass, 1985): (a) Idealised influence; (b) Inspirational motivation; (c) Individualised consideration; (d) Intellectual Stimulation. Idealised influence represents seeing the leader as a role model, thereby motivating the staff. Inspirational motivation represents leaders using the goals and sharing a vision to motivate the staff. Individualised consideration refers to leaders providing support to staff according to individuals' development needs, providing them with the greater opportunities to make decision, deal with challenges themselves, thereby helping them to achieve self‐actualization. When it comes to intellectual stimulation, this refers to leaders encouraging staff to solve difficult problems themselves and think critically, thereby helping them more creatively.

(Lussier & Achua, 2007).

Increasing nurses' OC is a major strategy to reduce the likelihood of nurses leaving their jobs (Meyer & Herscovitch, 2001; Somers, 2009; Takase et al., 2015). Therefore, it is important to identify the association between this style and nurses' OC. There are many studies exploring the relationships between leadership styles and employees' OC. Most studies found that there is a positive relationship between them (Dai et al., 2013; Luo et al., 2017; Nguni & Denessen, 2006; Rowden, 2000; Yahaya & Ebrahim, 2016; Yousef, 2000). However, most of the research has been conducted in business and manufacturing settings, with fewer studies focusing on nurses. Therefore, the previous findings are not sufficient to show that there is a positive association between the two variables in the nursing care setting. Moreover, a study suggested that nurse executives' TFL style negatively affected OC (Leach, 2005). Therefore, the relationship between the TFL style and nurses' OC is unclear. Notably, there has been no systematic review on the relationship between nurse managers' TFL style and nurses' OC. Therefore, we synthesised and analysed related studies (the literature was searched from January 2009 to December 2020) to identify the relationship between them.

### 2.1 Aims

The aim of this systematic review was to synthesise and analyse information from the available quantitative studies on the association between nurses' perceptions of their nurse managers' TFL style and nurses' OC. The review question was: What relationship between nurse managers' TFL style and nurses' OC?

### 2.2 Design

This study involved a quantitative systematic literature review, and the synthesis of the findings is presented in a narrative way.

### 2.3 Search methods

To search for relevant studies achieving comprehensiveness of coverage and maintaining the precision of the records retrieved, the search strategy based on the MeSH keywords (nurses, transformational leadership, organisational commitment) to search the text word (free text), including all spelling variants (e.g. UK and US spelling), synonyms, abbreviations, relevant truncation, and wildcard, to expand the range of search terms (McGowan et al., 2016). This research also used Boolean operators ‘OR’, and ‘AND’ to join the search term based on the logical relationship.

The search strategy in Table 1 was used to identify all relevant observational surveys published between January 2009 and December 2020 (to access the latest evidence) on CINAHL Complete, MEDLINE, PubMed, Business Source Complete, and Cochrane Library. In addition, it applied subject terms and English Language limitations in the search strategy.

**TABLE 1:** The search strategy.

PEO	Keywords	Search items	Search strategies
P (population)	Nurses	“Nurse*” OR “Healthcare worker*” OR “Nursing staff” OR “Nursing worker*”	((Nurse* OR Healthcare worker* OR Nursing staff OR Nursing worker*) AND (Transformational leadership OR TFl OR Transformational leader* OR Transformational manager*)) AND (Organisational commitment OR Personnel commitment OR Employee's commitment OR Affective Commitment OR Continuance Commitment OR Normative Commitment OR Commitment* OR Workplace commitment)
E (exposure factor)	Transformational leadership	“Transformational leadership” OR “TFL” OR “Transformational leader*”OR “Transformational manager*”	
O (outcomes)	Organisational Commitment	“Organisational commitment” OR “Personnel commitment” OR “Employee's commitment”OR “Affective Commitment” OR “Continuance Commitment” OR “Normative Commitment” OR “Commitment*” OR “Workplace commitment”	

Hooton et al. (2007) highlight that the published studies are likely to may tend to report effective findings compared with grey literature including the unpublished study in a systematic review can help to avoid the reporting bias. Therefore, to gain as much information as possible and to minimise the reporting bias, the researchers (XHH and HUB) also searched on some valid websites, such as OpenSIGLE (http://www.opengrey.eu/), to find the grey literature. To obtain as many studies as possible, the reviewers manually searched the reference lists of the eligible studies. In addition, to check that there were no similar reviews already underway, the reviewers also searched the PROSPERO systematic review database.

## 3 INCLUSION CRITERIA

### 3.1 Participants

As this review aimed to explore the relationship between TFL style and nurses' OC, the participants of interest were registered nurses.

### 3.2 Exposure factor

The nurses were exposed to nursing managers' TFL behaviour situations. The TFL style consists of idealised influence, inspirational motivation, individualised consideration, and intellectual stimulation (Bass, 1985). Therefore, the nurses in this review were exposed to the nurse managers' idealised influence, inspirational motivation, individualised consideration, and/or intellectual stimulation.

### 3.3 Outcomes

The primary outcomes of this review were nurses' OC, affective commitment (AC), continuance commitment (CC), or normative commitment (NC). As AC, CC, or NC to a particular organisation are encompassed by OC (Allen & Meyer, 1996; Mowday & Porter, 1979; Mowday & Steers, 1982), any study with results on OC, AC, CC, or NC were included in this review.

### 3.4 Types of studies

Only observational studies were included, as experimental trials were excluded owing to ethical issues. Sullivan and Garland (2013) suggest that an effective healthcare manager should adopt a suitable leadership style based on the specific situation. Clinical contexts are complex, so it is impossible to use only a single consistent type of leadership (Sullivan & Garland, 2013). Hill (2017) also highlights that combining using the situational leadership style allows nurse managers to adopt an appropriate style for different staff and dynamic environments, thereby motivating staff to work more effectively. Polgar and Thomas (2013) suggest that randomised controlled trials are not suited in certain complex situations. More specifically, it is not appropriate to force nurse managers in an intervention group to only use a TFL style and nurse managers in a control group to not use this style, as this would not allow them to take complex clinical situations into account. An inappropriate and ineffective leadership style negatively affects nurses' well‐being and the quality and safety of care, and it increases hospital costs (Al‐Hamdan et al., 2017; Cummings et al., 2010). Conducting an experimental trial may force nurse managers to adopt an inappropriate management style, which can harm the nurses, patients, and hospitals. This would violate the do no harm principle related to research participants, as detailed in Introduction to Research in the Health Sciences (Polgar & Thomas, 2013).

### 3.5 Language

In this systematic review, language was restricted to English.

### 3.6 Search outcome

The literature search was independently conducted by two reviewers (XHH and HHH). It yielded 80 studies (32 records from CINAHL Complete, 15 records from MEDLINE, 31 records from PubMed, seven from Business Source Complete, one records from Cochrane Library, zero from OpenGrey, and zero from PROSPERO).

### 3.7 Study selection

**FIGURE 1:**
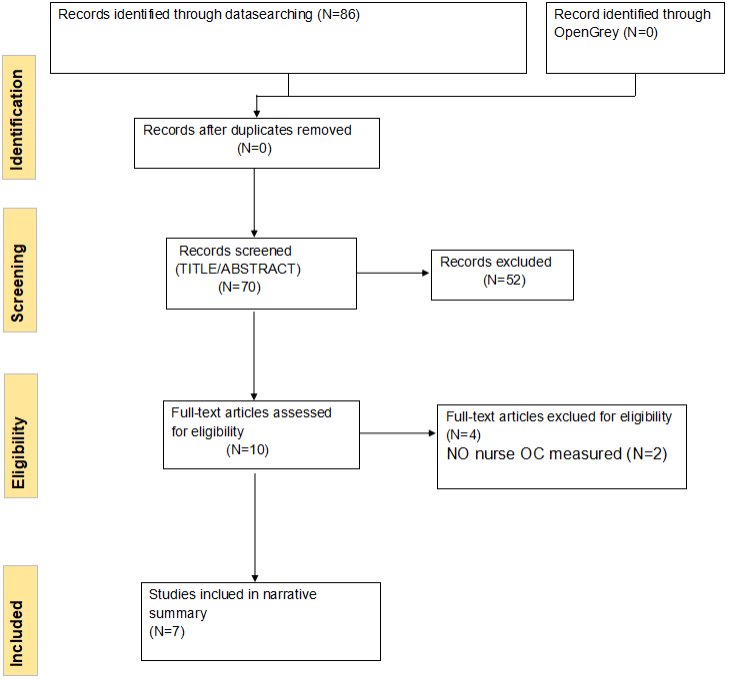
Preferred Reporting Items for Systematic Reviews and Meta‐Analyses (PRISMA) flowchart.

According to the Cochrane Handbook (Higgins & Green, 2011), this process is also best done by more than one author, which can help to reduce the risks of some eligible reports being discarded. Two reviewers (Xiong and Huang) independently conducted all study selection processes according to the selection criteria. When the agreement was not reached, other authors would be consulted. After removing duplicates in EndNote, 70 studies remained. After screening the titles and abstracts of the remained studies, there were 18 studies eligible for the criteria. After reading the full‐text versions of these 18 studies, seven were confirmed to meet the eligibility criteria, and the remaining 11 studies were excluded as seven of them cannot get the full text and the other four studies were not met the (PEO). No studies were identified based on manually searching the reference lists of the eligible studies. All these processes are shown in the Preferred Reporting Items for Systematic Reviews and Meta‐Analyses (PRISMA) flowchart (Shamseer et al., 2015) in Figure 1.

### 3.8 Quality appraisal

The Joanna Briggs Institute (JBI) Critical Appraisal Tool for Cross‐Sectional Studies is a useful quality assessment tool that is well‐accepted by users (Munn et al., 2014). The Critical Appraisal Checklist for Cross‐Sectional was applied to assess the bias, validity, limitations, and generality of cross‐sectional studies (Munn et al., 2014). Therefore, this tool was used to assess the included studies' quality. Two independent reviewers (Xiong and Huang) conducted the critical appraisal and reached a consensus. When no consensus was reached, a third author would be consulted.

### 3.9 Data extraction

To reduce the error rate (Higgins & Green, 2011), two reviewers (XHH and HH) transparently and independently extracted the following data standardised data extraction tables developed in a pilot study: first author, year of publication, aims, study design; sample characteristics (sample selection; sample No.), study setting/context, measurement tools, reliability of the measurement tools, statistical analysis methods, and outcomes. When no consensus was reached, a third author would be consulted.

### 3.10 Synthesis

The included studies exhibited considerable clinical, methodological, and statistical heterogeneity. For example, the participants in four studies were all types of nurses (Al‐Yami & Watson, 2018; Brewer et al., 2016; Kodama et al., 2016; Lin et al., 2015), while the studies by Al‐Hussami (2009), Al‐Yami and Watson (2018), and Xie et al. (2020) focused on only nursing home nurses, acute nurses, and aged care agency nurses, respectively, which caused clinical heterogeneity. Additionally, there were various sampling methods and study designs, and there were seven tools to assess TFL style, six tools to assess OC, and seven combinations of statistical methods to analyse the data, which caused large methodological and statistical heterogeneity. Higgins and Green (2011) suggest that the data synthesis in a systematic review should involve a narrative approach when the data cannot be combined or when the data exhibits large heterogeneity, because of the high risk of reaching a misleading conclusion when conducting a meta‐analysis in cases involving large heterogeneity. Therefore, the data synthesis in this review involved a narrative approach.

## 4 RESULTS

### 4.1 Characteristics of included studies

Seven cross‐sectional surveys were included in this review (Al‐Hussami, 2009; Al‐Yami & Watson, 2018; Asiri et al., 2016; Brewer et al., 2016; Kodama et al., 2016;Lin et al., 2015 ; Xie et al., 2020). They all aimed to explore the relationship between TFL style and nurses' OC. However, the sample selection and study design varied. The samples in the studies by Al‐Hussami (2009), Lin et al. (2015), Asiri et al. (2016), and Al‐Yami and Watson (2018) were random but the former two studies used stratified random samples while the latter two did not. In contrast, the samples in the studies by Brewer et al. (2016), Kodama et al. (2016), and Xie et al. (2020) were convenience samples rather than random samples. Moreover, the study by Brewer et al. (2016) is a cross‐sectional study that was part of a 10‐year longitudinal panel study.

This review involved 2885 nurses, almost half of these participants (*n* = 1037) belonged to the study by Brewer et al. (2016). The study with the smallest sample size (*n* = 60) was by Al‐Hussami (2009). The sample sizes in the other five studies by Lin et al. (2015), Kodama et al. (2016), Asiri et al. (2016), Al‐Yami and Watson (2018), and Xie et al. (2020) were 651, 369, 332, 219, and 217, respectively (Table 2). Although all samples were nurses, they were from different nursing professions. The samples of Al‐Hussami (2009)'s and Xie et al. (2020)'s were aged care nurses; the samples of the Kodama et al. (2016)'s and Asiri et al. (2016)'s were acute nurses; the samples of the other three studies were different nursing professions (general, paediatric, dental, diabetic, Nursing home, etc).

**TABLE 2:** Included study characteristics (Summary).

Author (year)	Aim	Study design; sample selection; sample No.	Setting	Measures	Reliability Cronbach's α	Analysis	Results
Al‐Hussami (2009)	To assess the associations of job satisfaction, perceived organisational support, TFL behaviour and education level with OC	Cross‐sectional survey; Stratified random sample; *N* = 60	Southeastern USA; 4 nursing homes; Nursing homes nurses	MLQ (Bass & Aviolo, 1992) OCQ (Meyer et al., 1993)	0.79–0.85 0.85	Pearson's correlation and Multiple linear regressions	Positive: Pearson correlation *r* (55) = 0.71, *p* ≤ 0.05; Multiple linear regressions:Coefficients *B* = 0.102 SE = 0.111 *β* = 0 0.056 *t* = 0.911 *p* = 0.366
Lin et al. (2015)	To assess the associations of TFL and nurses' mental health with OC and job satisfaction	Cross‐sectional survey; Convenience sample; *N* = 651	China; 12 hospitals; Nurses	MLQ (Bass & Avolio, 1994) OCQ (Mowday & Porter, 1979)	0.975 0.878	Pearson's correlation and ANOVA	Positive: Pearson correlations *r* = 0.321, *p* ≤ 0.01; Analysis of variance (ANOVA) *β* = 0.50, *p* < 0.05
Brewer et al. (2016)	To assess the associations of TFL with early career nurses' intent to stay, job satisfaction, and OC	Cross‐sectional survey with 10‐year longitudinal design; Convenience samples; *N* = 1037	US; Hospitals across the country; Nurses	TFLS (Hammer et al., 2012) OCS (Price, 2001)	0.917 0.863	Correlations analysis and Ordered probit model (Greene, 2008)	Positive: Correlations analysis *r* = 0.495, *p* ≤ 0.01; Ordered probit model Coef. = 0.098 SE = 0.03 *p* > z = 0.001 ME = 0.010
Kodama et al. (2016)	To assess the associations of nurse managers' leadership with nurses' AC	Cross‐sectional survey; Convenience sample; *N* = 369	Japan; fourmid‐sized acute care hospitals acute nurses;	MLQ (Avolio & Bass, 2004) ACQ (Shibaoka et al., 2010)	0.72–0.87 0.70–0.95	Multiple logistic regression	Positive: Multiple logistic regression *β* = 0.80 SE = 0.27 OR = 2.23 95% CI (1.31–3.80) ref *p* ≤ 0.0032
Asiri et al. (2016)	To assess the associations among leadership, empowerment, and OC	Cross‐sectional survey; Random samples; *N* = 332	Saudi Arabia; 1 hospital;a single health care institution acute nurses	MLQ (Avolio & Bass, 2004) OCS (Allen & Meyer, 1996)	0.94 0.79	Pearson's correlation and stepwise linear regression	Negative: Pearson Correlation *r* = −0.113, *p* ≤ 0.045; Stepwise linear regression B = 0.14 SE = 0.08 *t* = 1.8 *p* ≤ 0.074
Al‐Yami et al. (2018)	To assess the associations of nurse managers' leadership styles and nurses' OC	Cross‐sectional survey; Random samples; *N* = 219	Saudi Arabia; 2 complex hospitals; Nurses	MLQ (Bass, 1985) OCQ (Mowday & Porter, 1979)	>0.60 0.77	Pearson's correlation and hierarchical regression	Positive: Pearson Correlation *r* = 0.432, *p* ≤ 0.01; Hierarchical regression analysis *B* = 7.0 SE = 0.35 *β* = 0,35 *t* = 5.1 *R* ^2^ = 0 0.25 *p* ≤ 0.05
Xie et al. (2020)	To assess the associations of TFL and work culture on nurses' willingness to stay in the aged care industry.	Cross‐sectional survey; Convenience sample; *N* = 217	China; 1 aged care agency Nursing staff	TFLQ (Chang and Lee, 2007) OCQ (Sjöberg and Sverke,2000)	0.928 0.899	Structural equation modelling analyses	Positive: *β* = 0.345, *p* < 0.01

The participants worked in the USA (Al‐Hussami, 2009; Brewer et al., 2016), Saudi Arabia (Al‐Yami & Watson, 2018; Asiri et al., 2016), China (Lin et al., 2015; Xie et al., 2020), and Japan (Kodama et al., 2016) (Table 2). Brewer et al. (2016)'s samples were nursing staff from a whole country's different hospitals; Al‐Hussami (2009)'s samples were nursing staff from 12 different hospitals. Al‐Hussami (2009)'s and Kodama et al. (2016)'s samples were from four different hospitals; Al‐Yami and Watson (2018)'s samples were from two different hospitals; Asiri et al. (2016)'s and Xie et al. (2020)'s samples were from one medical institution. Thus, the participants in this review included participants that worked in western countries and others that worked in eastern countries.

### 4.2 Tools to evaluate TFL style

Seven tools were used to evaluate the exposure factor, that is, nurses' perceptions of their nurse managers' TFL style (Table 2). Al‐Hussami (2009) used the Multifactor Leadership Questionnaire (MLQ) Form 6 S (Bass & Aviolo, 1992) (Cronbach's *α*: 0.79–0.85). Lin et al. (2015) used the Multifactor Leadership Questionnaire (MLQ) (Bass & Avolio, 1994) (Cronbach's *α*: 0.975). Brewer et al. (2016) used the 6‐item Transformational Leadership Scale (TFLS) (Hammer et al., 2012) (Cronbach's *α*: 0.917), which is based on a scale developed by Podsakoff et al. (1990). Kodama et al. (2016) used the Multifactor Leadership Questionnaire (MLQ) Form 5X‐Short (Avolio & Bass, 2004) (Cronbach's *α*: 0.72–0.87). Asiri et al. (2016) used the Multifactor Leadership Questionnaire (MLQ) (Avolio & Bass, 2004) (Cronbach's *α*: 0.94). Al‐Yami and Watson (2018) used the Multifactor Leadership Questionnaire (MLQ) (Bass, 1985) (Cronbach's *α*: >0.6). Xie et al. (2020) used the Transformational.

Leadership Questionnaire (TFLQ) (Chang and Lee, 2007) (Cronbach's *α*: 0.928).

### 4.3 Tools to evaluate OC

Six tools were used to evaluate the outcome, that is, nurses' OC (Table 2). Al‐Hussami (2009) used the organisational Commitment Questionnaire (OCQ) developed by Meyer et al. (1993) (Cronbach's *α*: 0.85). Lin et al. (2015) and Al‐Yami and Watson (2018) used the organisational Commitment Questionnaire (OCQ) developed by Mowday and Porter (1979) (Cronbach's *α*: 0.878 and 0.77, respectively). Brewer et al. (2016) used the organisational Commitment Scale (OCS) developed by Price (2001) (Cronbach's *α*: 0.863). Kodama et al. (2016) used the organisational Justice Questionnaire of Affective Commitment (ACQ) developed by Shibaoka et al. (2010) (Cronbach's *α*: 0.70–0.95). Asiri et al. (2016) used the organisational Commitment Scale (OCS) developed by Allen and Meyer (1996) (Cronbach's *α*: 0.79). Xie et al. (2020) used the organisational Commitment Questionnaire (OCQ) developed by Sjoberg and Sverke (2000) (Cronbach's *α*: 0.899).

### 4.4 Summary of the statistical analysis methods

The statistical methods varied among the seven studies (Table 2). Al‐Hussami (2009) used Pearson's correlation and multivariate linear regression. Lin et al. (2015) used Pearson's correlation and analysis of variance (ANOVA). Brewer et al. (2016) used correlation analysis and an ordered probit model (Greene, 2008). Kodama et al. (2016) used multivariate logistic regression. Asiri et al. (2016) used Pearson's correlation and stepwise multivariate linear regression. Al‐Yami and Watson (2018) used Pearson's correlation and hierarchical regression. Lastly, Xie et al. (2020) used structural equation modelling. The statistical tests (regression or other analyses) in each included study all indicated a good fit with the data.

### 4.5 Summary of study outcomes and data synthesis

All included studies demonstrate that there is a correlation between the transformational leadership style and nurses' organisational commitment. Six studies are finding that the transformational leadership style has a positive effect on nurses' organisational commitment (Al‐Hussami, 2009; Al‐Yami & Watson, 2018; Brewer et al., 2016; Kodama et al., 2016; Lin et al., 2015). only Asiri et al. (2016) found that the association of the TFL style with nurses' OC in acute care units in Saudi Arabia was negative using Pearson's correlation analysis (*r* = − 0.113, *p* ≤ 0.045), but was positive using Stepwise linear regression (*B* = 0.14 SE = 0.08 *t* = 1.8 *p* ≤ 0.074), although not significant. All the information were present in Table 2.

The result of the quality assessment from JBI Cross‐sectional Critical Appraisal Tools of included articles is in Table 3. Brewer et al. (2016)'s study was considered high‐quality evidence because eight‐question of the JBI Cross‐sectional Tools were been considered in this study. Compared with these two studies, Al‐Yami and Watson (2018)'s study was considered as low quality of evidence, because it found that two question JBI Cross‐sectional Tools were unclear, and the exposure was not measured validly and reliably. The other five studies of the review were considered moderate‐quality evidence because one or two questions JBI Cross‐sectional Tools were unclear.

**TABLE 3:** Summary quality assessment.

The summary of +A1:H11 the quality assessment of the included study using JBI cross‐sectional critical appraisal tools							
Studies ID questions	Al‐Hussami (2009)	Lin et al. (2015)	Brewer et al. (2016)	Kodama et al. (2016)	Asiri et al. (2016)	Al‐Yami et al. (2018)	Xie et al., 2020
1. Were the criteria for inclusion in the sample clearly defined?	Unclear	Yes	Yes	Yes	Unclear	Unclear	Unclear
2. Were the study subjects and the setting described in detail?	Yes	Yes	Yes	Yes	Yes	Yes	Yes
3. Was the exposure measured in a valid and reliable way?	Yes	Yes	Yes	Yes	Yes	No	Yes
4. Were objective, standard criteria used for measurement of the condition?	Yes	Yes	Yes	Yes	Yes	Yes	Yes
5. Were confounding factors identified?	Yes	Yes	Yes	Yes	Yes	Yes	Yes
6. Were strategies to deal with confounding factors stated?	Yes	Yes	Yes	No	Unclear	Unclear	Unclear
7. Were the outcomes measured in a valid and reliable way?	Yes	Yes	Yes	Yes	Yes	Yes	Yes

## 5 DISCUSSION

Nurse shortages are a global issue and ageing populations will worsen the existing issues. The high turnover rates among nurses contribute to nursing shortages. Training and recruiting more nursing students cannot fully address this problem. More effort should be made to retain nurses. A major factor underlying the retention rate is nurses' organisational commitment, which is related to effective leadership. The transformational leadership style is the prevailing contemporary leadership style among nurse managers. However, it is controversial whether there is a positive or negative association between transformational leadership style and nurses' organisational commitment. Therefore, we conducted this review to find evidence to explore the relationship between transformational leadership style and nurses' organisational commitment, thereby providing some suggestions for nursing managers to adopt an appropriate leadership style and improve nurses' organisational commitment. Meanwhile, it can help the hospital reduce the cost of nurses turnover and recruit new nurses. Moreover, addressing the nursing shortage can help to deal with the ageing population's healthcare problem in the long term.

This review found seven studies met the included criteria. All included studies found that there was an association between the TFL style and nurses' OC. Six studies reported that the TFL style had a positive effect on nurses' OC (Al‐Hussami, 2009; Al‐Yami & Watson, 2018; Brewer et al., 2016; Kodama et al., 2016; Lin et al., 2015; Xie et al., 2020). Only one study found that the association of the TFL style with nurses' OC was negative using Pearson's correlation analysis, but was positive using Stepwise linear regression, although not significant (Asiri et al., 2016).

Asiri et al. (2016)'s samples were only from an acute care unit in Saudi Arabia, the results may only be generalizable to this unit or acute care nurses in Saudi Arabia. However, this negative finding was contrary to the other positive finding (Kodama et al., 2016) in this review which also studied the acute nurses, but in Japan. These different results may be influenced by different cultural backgrounds. Furthermore, the study (Asiri et al., 2016) reported that the mean score of the nurses' perceptions of their nurse managers' TFL level was 2.55 (SD = 0.75) (Asiri et al., 2016). However, Bass and Avoliov (1997) reported that an effective TFL style required all component scores to be >3.0. This indicates that the nurse managers' TFL style in the study by Asiri et al. (2016) was not effective. Al‐Hamdan et al. (2017) and Aboyassin and Abood (2013) highlight that ineffective leadership styles negatively affect employees' OC. Therefore, the finding by Asiri et al. (2016) that there was a negative association between the TFL style and nurses' OC may have occurred because the nurse managers' TFL style was ineffective. Moreover, Asiri et al. (2016)'s study was considered a piece of moderate‐quality evidence (Table 3). Therefore, further research is required to support this conclusion, due to the lack of primary studies or systematic reviews.

There were six positive findings on the association between the TFL style and nurses' OC in this review. Two positive findings were focused on aged care nurses (Al‐Hussami, 2009; Xie et al., 2020). Both of them were moderate‐quality evidence (Table 3). Al‐Hussami (2009) had a small original sample size (only 60 participants were enrolled) and all the participants were four nursing home nurses working in the US. Xie et al. (2020) had 217 aged care nurses from one aged care agency. These two studies consistently found that the TFL style had a positive association with aged care nurses' OC.

The other three positive findings were from nurses from different nursing professions (general, paediatric, dental, diabetic, Nursing home, etc). One high‐quality piece of evidence (Table 3) (Brewer et al., 2016) involved 1034 nurses from various US hospitals and various nursing professions who had been licensed for 7.5–8.5 years, the large number of participants from different hospitals across the whole of the US strengthens the study. One moderate‐quality evidence (Table 3) (Lin et al., 2015) involved 651 nurses from 12 China hospitals and various nursing professions, the adequate sample size (this study was the second‐largest study), and the scientific sampling method make this study was strong. The other low‐quality evidence (Table 3) (Al‐Yami & Watson, 2018) involved 219 nurses from various nursing professions in two hospitals in Saudi Arabia. These three studies consistently found that the TFL style had a positive association with the nurses' OC. Consequently, these findings can be generalisable to China, Saudi Arabia, and the US. In addition, these threes studies' samples also included aged care nurses, though it cannot be known the exact percent for this group. Therefore, these three studies can also generalisable to aged care nurses.

The remaining study (Kodama et al., 2016) found that the TFL style had a positive association with acute nurses*'* OC in Japan. This moderate‐quality evidence (Table 3) involved 369 acute nurses. However, there was one study in this review (Asiri et al., 2016) found that the TFL style had a negative association with acute nurses' OC in Saudi Arabia. As mentioned above, further research is needed to address this controversial problem, due to the lack of sufficient evidence.

### 5.1 Limitations

This study has several limitations. First, all included studies are cross‐sectional surveys; hence, the limitations of cross‐sectional surveys contribute to the limitations of this systematic review. A limitation of cross‐sectional surveys is that they only allow each participant to be assessed once, so changes over time cannot be assessed (Polgar & Thomas, 2013). However, OC is developed over a long time (Gao‐Urhahn & Jaros, 2016). The change process regarding nurses' OC cannot be recorded by cross‐sectional studies, though it can be recorded by longitudinal studies. Therefore, the cross‐sectional design of the included studies increases the risk of bias in this review.

Second, the exposure factor and the outcome were assessed using self‐administered questionnaires in all included studies. There is a risk that a participant may not report the actual behaviours witnessed or their individual experiences and feelings (Polgar & Thomas, 2013), for example, they may misunderstand a question or feel pressure from their nurse manager to answer in a certain way. This increases the risk of bias in this review. Additionally, as all the measurement tools assessed the participants ‘perceptions of the nurse managers’ TFL level or their feelings about OC, the answers were all subjective rather than objective. In addition, Al‐Yami and Watson (2018) used the MLQ (Bass, 1985) to assess the outcome, and the MLQ has questionable validity, which negatively affected the study validity.

Third, the sampling methods used in the included studies also contribute to the limitations of this review (Bettany‐Saltikov, 2012). Brewer et al. (2016), Xie et al. (2020), and Kodama et al. (2016) used convenience samples rather than random samples. Although Al‐Hussami (2009) used a stratified random sample, the study had the smallest sample size (*n* = 60), and only nursing home nurses were assessed. In addition, Asiri et al. (2016) only obtained their random sample from a single acute care unit, which negatively affected the results' generalisability. Moreover, the studies by Al‐Hussami (2009), Asiri et al. (2016), Xie et al. (2020), and Al‐Yami and Watson (2018) had unclear inclusion and exclusion criteria. Furthermore, Al‐Hussami (2009) did not discuss why five participants' data were missing. All these factors also contribute to bias in this review.

Fourth, the studies by Al‐Hussami (2009) and Asiri et al. (2016) did not provide details of research ethics committee approvalor participant informed consent, which increases the risk of ethical issues in this review.

### 5.2 Strengths of the review

The strengths of this review include involving a total of 2885 participants. The huge sample size contributes to the generalizability of this review. Moreover, this review included studies conducted in varied settings: three involved western countries (Al‐Hussami, 2009; Brewer et al., 2016; Kodama et al., 2016) and four involved eastern countries (Al‐Yami & Watson, 2018; Asiri et al., 2016; Lin et al., 2015; Xie et al., 2020). This contributes to the generalisability of this review to both western and eastern countries.

## 6 CONCLUSION

In summary, all the included studies found there is an association between the TFL style and nurses' OC. Six studies consistently demonstrated that the TFL style was positively associated with nurses' OC, which is consistent with other studies in business (Dai et al., 2013; Luo et al., 2017). Only one study reported that the relationship between these two variables was negative in Pearson's correlation analysis, but was not significant positive in Stepwise linear regression; moreover, this study is not a strong evidence, and the finding may be due to the nurse managers' ineffective TFL style in the acute care unit or the culture influence. A further research is needed to address the whether the negative findings may be due to the nurse managers' ineffective TFL style or he culture influence.

## AUTHOR CONTRIBUTIONS

All authors have agreed on the final version and meet at least one of the following criteria [recommended by the ICMJE (http://www.icmje.org/recommendations/)]:

• substantial contributions to conception and design, acquisition of data, or analysis and interpretation of data;

• drafting the article or revising it critically for important intellectual content.

## FUNDING STATEMENT

None.

## CONFLICT OF INTEREST STATEMENT

No conflict of interest has been declared by the authors.

## Data Availability

Data openly available in a public repository that issues datasets: Asiri et al., (2016); Al‐Yami & Watson, (2018); Al‐Hussami (2009); Brewer et al., (2016); Kodama et al., (2016); Lin et al., (2015); Xie et al. (2020).
